# A Rare Case of Bilateral Mesiodens With Dens Invaginatus Obstructing Permanent Incisor Eruption

**DOI:** 10.1002/ccr3.71452

**Published:** 2025-11-16

**Authors:** Hanane Pourheidary

**Affiliations:** ^1^ Department of Periodontology Shahed Dental School, Shahed University Tehran Iran

**Keywords:** cone‐beam computed tomography, dens invaginatus, diagnosis, impacted maxillary central incisors, mesiodens, multidisciplinary approach, pediatric dentistry, supernumerary teeth

## Abstract

The co‐occurrence of mesiodens and dens invaginatus (DI) is very uncommon. Such anomalies may impede the eruption of permanent maxillary central incisors and can appear radiographically similar to normally developing teeth. An 11‐year‐old girl presented with failure of eruption of the permanent maxillary central incisors. Panoramic radiography and cone‐beam computed tomography (CBCT) revealed two palatobuccally positioned supernumerary mesiodens, both of which exhibited DI. Surgical extraction of the mesiodens was performed, and guided bone regeneration (GBR) was carried out to preserve the alveolar ridge and optimize conditions for subsequent eruption and orthodontic treatment. At the time of reporting, the permanent incisors remain unerupted. Delay in intervention led to loss of mesiodistal space as adjacent teeth drifted. Advanced orthodontic treatment, including distalization, is currently in progress. This case underscores that prompt diagnosis using CBCT and a coordinated multidisciplinary approach is crucial for managing complex dental anomalies and ensuring favorable outcomes.

**Summary**

- Bilateral mesiodens with DI is extremely rare and poses significant diagnostic challenges.
- Early detection and CBCT imaging are critical for accurate diagnosis, and timely multidisciplinary intervention helps prevent impaction and guides proper eruption of the permanent incisors.

## 1 Introduction

Mesiodens, a type of supernumerary tooth located in the midline of the maxilla, is uncommon, with a prevalence ranging from 0.15% to 1.9% [1]. Cases involving multiple mesiodens, especially those associated with other dental anomalies like dens invaginatus (DI), are exceedingly rare [2]. DI, sometimes referred to as “tooth within a tooth,” is a developmental anomaly where the enamel and dentin fold inward into the pulp cavity during tooth development, leading to potential complications such as delayed or blocked tooth eruption, impaction, or misalignment [3].

This case report outlines the diagnostic steps, imaging findings, and surgical management of two mesiodens with DI, which obstructed the eruption of the permanent central incisors in a pediatric patient. The case highlights the importance of a multidisciplinary approach, including the use of three‐dimensional (3D) imaging and guided bone regeneration, in effectively managing complex dental anomalies [4, 5].

## 2 Case History/Examination

An 11‐year‐old girl presented with concerns about the delayed eruption of her permanent maxillary central incisors. She had no relevant medical or family history, and there were no prior dental extractions, trauma, or orthodontic treatments.

**FIGURE 1:**
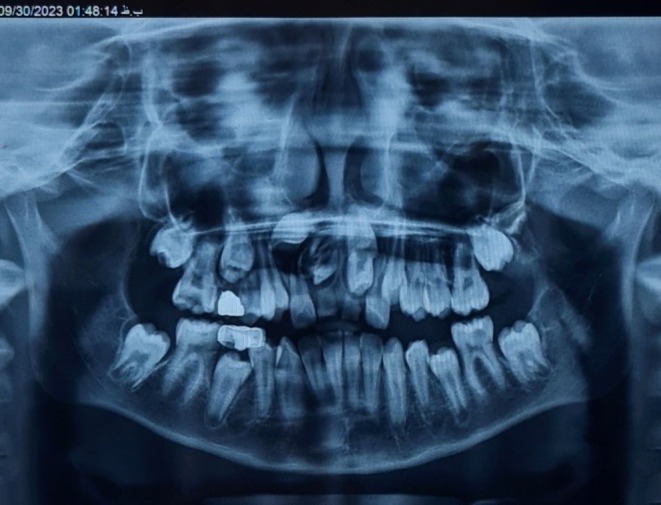
Panoramic radiograph showing unerupted permanent maxillary central incisors obstructed by two supernumerary teeth (mesiodens) located in the anterior maxilla. The image provided the first indication of an eruption disturbance, prompting further 3D imaging.

Clinical examination revealed that the primary maxillary central incisors were still present and immobile, suggesting an underlying obstruction to the eruption of the permanent teeth. A panoramic radiograph revealed two unerupted mesiodens in the anterior maxillary region, positioned between the roots of the primary central incisors, obstructing the eruption path of the permanent incisors (Figure 1).

**FIGURE 2:**
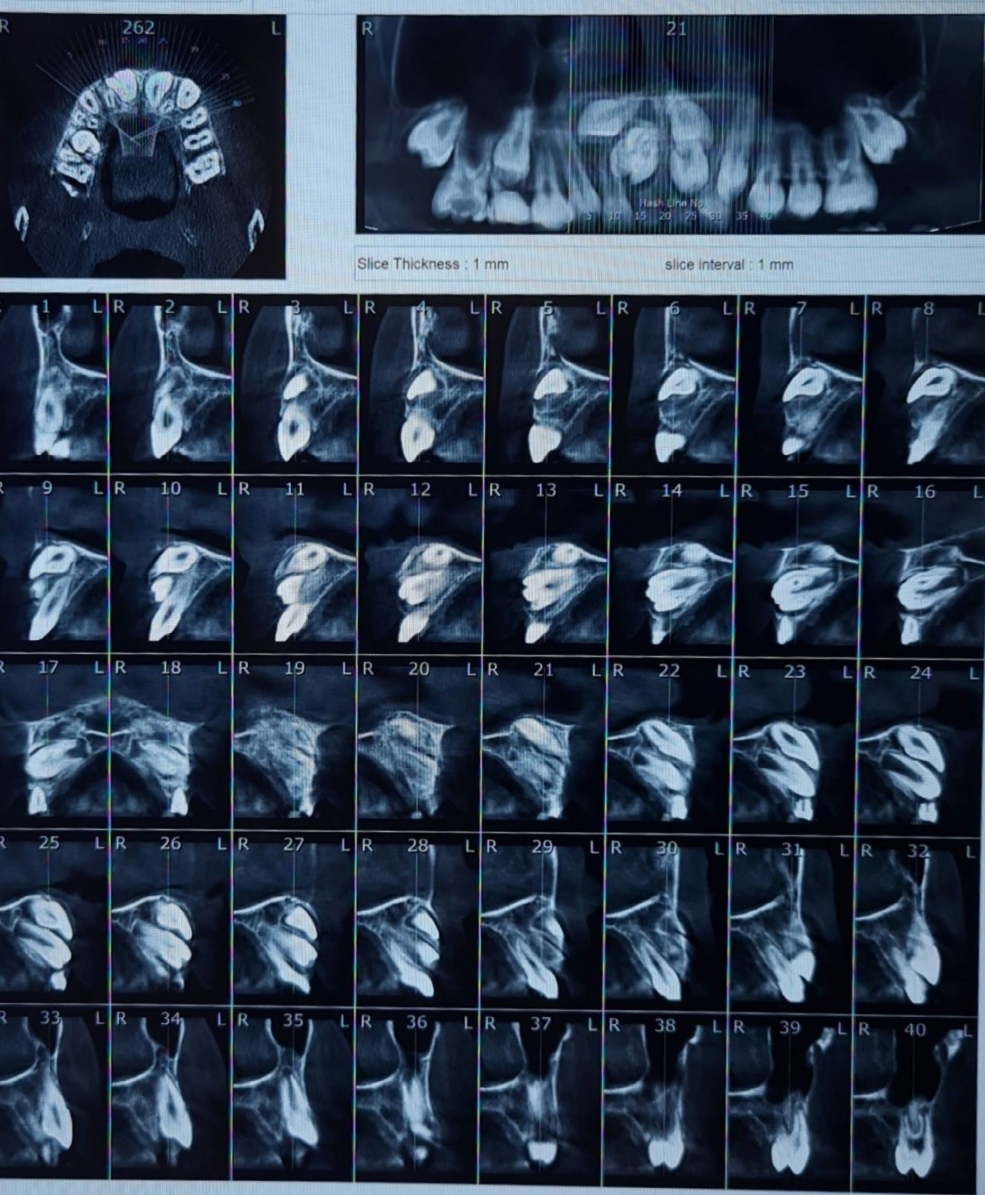
CBCT (axial, coronal, sagittal) demonstrates enamel‐lined invagination within each supernumerary tooth (mesiodens), consistent with DI, and their 3D relationship to the unerupted incisors. These findings—indistinct on panoramic imaging—identify the precise obstruction pathway and invagination depth (Oehlers's classification), directly informing surgical timing and approach.

A cone‐beam computed tomography (CBCT) scan was then performed, providing detailed 3D information about the relationship between the mesiodens, the permanent central incisors, and surrounding bone structures (Figure 2). The mesiodens were positioned in close proximity to the crowns of the permanent incisors in a palatobuccal direction, and both exhibited DI, adding complexity to the case [5, 6].

## 3 Surgical Procedure

A treatment plan was developed for the surgical removal of the mesiodens to facilitate the eruption of the permanent central incisors. Local anesthesia was administered using anterior superior alveolar (ASA) and nasopalatine nerve blocks, achieved with 2% lidocaine and 1:100,000 epinephrine [7].

**FIGURE 3:**
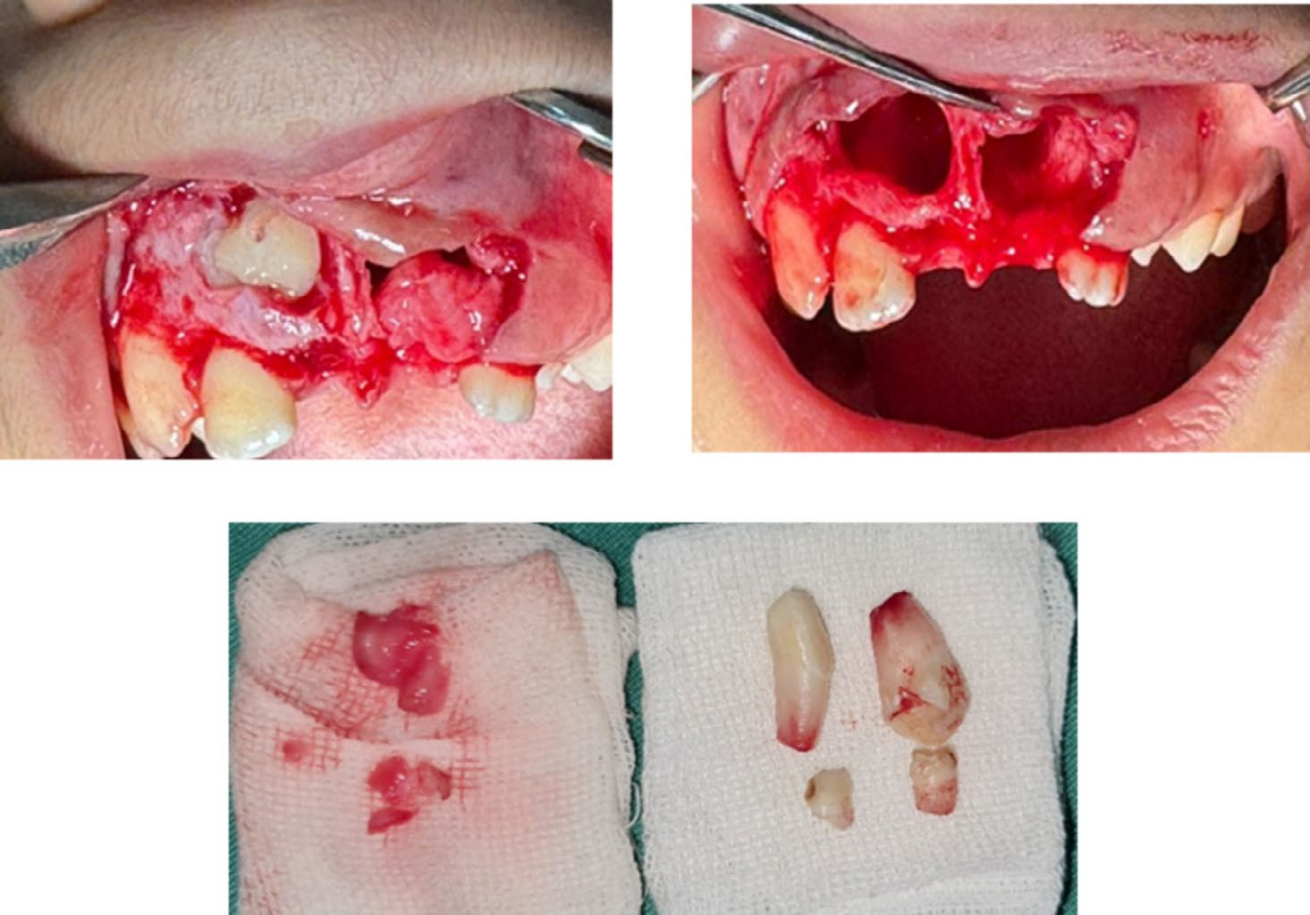
Intraoral clinical view of the exposed mesiodens following surgical flap elevation. The anomalous crown morphology confirmed the preoperative radiographic findings and guided extraction.

A submarginal mucoperiosteal flap was raised using a buccal approach, based on CBCT findings showing thinner buccal cortical bone in this area. After elevating the flap and removing the primary central incisors, the buccal cortical bone was removed to expose the mesiodens. Both mesiodens were extracted without complications (Figure 3) [8, 9, 10].

**FIGURE 4:**
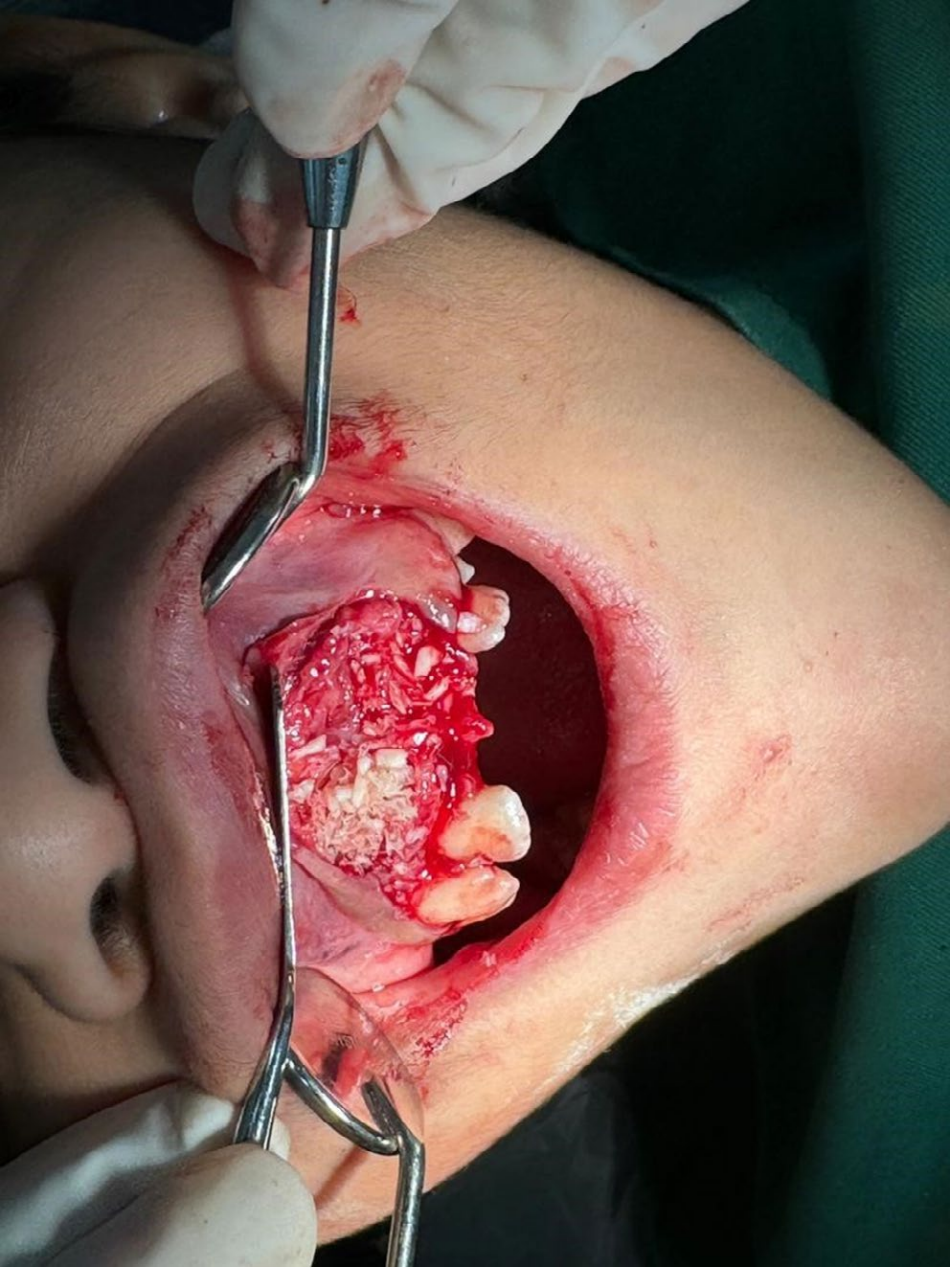
Postextraction view demonstrating guided bone regeneration (GBR) at the extraction site to preserve alveolar ridge integrity and optimize conditions for the eruption of permanent incisors.

Guided bone regeneration (GBR) was performed to maintain space and support the healing process, as the defect left by the mesiodens extraction raised concerns about soft tissue collapse (Figure 4) [7].

The surgical site was sutured with 4/0 polyglycolic acid (PGA) thread, and postoperative care instructions were provided. Follow‐up visits were scheduled to monitor healing and the eruption of the permanent central incisors [8, 11].

## 4 Conclusion and Results (Outcome and Follow‐Up)

At follow‐up, satisfactory healing was observed at the surgical site. Although spontaneous eruption of the permanent incisors had not yet occurred at the time of writing, the procedure successfully prevented complications such as root resorption, cyst formation, and adjacent tooth displacement.

The case illustrates the importance of early detection and multidisciplinary management, incorporating CBCT imaging, surgical precision, and guided bone regeneration to achieve optimal outcomes in complex dental anomalies.

## 5 Discussion

This case report presents an exceptionally rare occurrence of bilateral mesiodens associated with DI that obstructed the eruption of the permanent maxillary central incisors. Mesiodens, the most common type of supernumerary tooth, has a prevalence ranging from 0.15% to 1.9%, while DI in permanent teeth varies from 0.3% to 10%, typically affecting lateral incisors but also found in other teeth, including supernumerary teeth [12, 13].

The combination of DI in mesiodens, especially bilateral cases, is exceptionally rare and has been documented mainly in isolated case reports or small case series.

### 5.1 Radiographic Challenges

Existing reports describe DI in supernumerary teeth as a common cause of eruption disturbances. The rarity of this combination emphasizes the need for heightened suspicion when anterior supernumeraries exhibit unusual internal anatomy on CBCT. Early and accurate diagnosis using advanced imaging techniques like CBCT is essential to avoid misdiagnoses and prevent potential complications [1, 2].

### 5.2 Why This Combination Is Harder Than Simple Mesiodens

Panoramic radiographs often show bulbous crowns in anterior supernumerary teeth, which may resemble developing permanent incisors. The internal morphology of these teeth is often ambiguous, making them difficult to differentiate from normal developing teeth. In our case, the panoramic radiograph raised suspicion, but the CBCT provided detailed 3D information that clarified the diagnosis by revealing an enamel‐lined tract within the mesiodens, which is diagnostic of DI. This allowed precise determination of the 3D position of the mesiodens, their relationship to the unerupted incisors, and the nasopalatine canal, which would have been unclear or missed on panoramic radiographs [14].

### 5.3 Prevalence and Clinical Significance

Supernumerary teeth, particularly mesiodens, are extremely rare. Bilateral cases with DI are sporadically reported in the literature. These anomalies often mimic the development of permanent incisors on two‐dimensional radiographs, making accurate diagnosis difficult. CBCT imaging plays a crucial role by clearly depicting enamel‐lined invaginations and atypical canal morphology, which are not visible on conventional radiographs. Early identification through CBCT allows timely intervention, preventing eruption disturbances and minimizing orthodontic complications [10, 12].

### 5.4 Timing and Eruption Management

At the time of presentation, the patient was 11 years old. Both panoramic and CBCT radiographs indicated that bilateral mesiodens with DI obstructed the eruption of the permanent maxillary central incisors, with adjacent tooth drift causing mesiodistal space loss.

Contemporary guidelines suggest that obstructive mesiodens should ideally be removed during the mixed dentition phase (around 7 years) to maximize the likelihood of spontaneous eruption and minimize the need for complex orthodontic treatment. However, in this case, the mesiodens were not removed until the patient was 11 years old, resulting in delayed eruption and the need for advanced orthodontic treatment, including distalization of adjacent teeth to regain lost space [7].

This case underscores the importance of timely diagnosis and early intervention. Delayed removal necessitated more invasive orthodontic procedures and prolonged treatment duration. While spontaneous eruption can still occur in cases of delayed mesiodens removal, it may be significantly delayed (6–36 months), as seen in this case. Early intervention would have minimized the need for complex orthodontic treatment and improved the overall prognosis [7, 15].

## 6 Conclusion

This case highlights the rare co‐occurrence of bilateral mesiodens with DI and the importance of early diagnosis using CBCT imaging. CBCT allows for accurate visualization of complex dental anomalies, playing a key role in treatment planning. Timely removal of mesiodens is critical to ensuring favorable outcomes, as early intervention reduces the risk of space loss, impaction, and the need for advanced orthodontic treatments. A multidisciplinary approach is essential for optimizing clinical outcomes and minimizing complications in cases like this, which are rare and complex.

## 7 Clinical Implications

- CBCT imaging is crucial for diagnosing DI in mesiodens, allowing for precise visualization and diagnosis of subtle anomalies.
- Early surgical management is vital in preventing complications like root resorption, cyst formation, and adjacent tooth displacement.
- A multidisciplinary approach—involving radiology, surgery, GBR specialists, and orthodontics—is essential for the effective management of complex dental anomalies.

## 8 Limitations and Future Directions

- A key limitation is the lack of documented spontaneous eruption during the follow‐up period.
- Longitudinal CBCT‐based studies or case series are recommended to evaluate the prevalence, morphological diversity, and optimal timing for surgical intervention in similar cases [10, 16, 17].

## Author Contributions

**Hanane Pourheidary:** conceptualization, data curation, investigation, methodology, project administration, validation, visualization, writing – original draft, writing – review and editing.

## Consent

The treatment procedure and potential surgical complications were explained, and written informed consent was obtained from the patient's parent for both the treatment and the publication of this case report and accompanying clinical images.

## Data Availability

All data generated or analyzed during this case report is included in this published article.
